# Room Temperature Radiolytic Synthesized Cu@CuAlO_2_-Al_2_O_3_ Nanoparticles

**DOI:** 10.3390/ijms130911941

**Published:** 2012-09-20

**Authors:** Alam Abedini, Elias Saion, Farhad Larki, Azmi Zakaria, Monir Noroozi, Nayereh Soltani

**Affiliations:** Department of Physics, Faculty of Science, Universiti Putra Malaysia, Serdang, Selangor 43400, Malaysia; E-Mails: elias@science.upm.edu.my (E.S.); farhad.larki@gmail.com (F.L.); azmizak@science.upm.edu.my (A.Z.); monir_noroozi@yahoo.com (M.N.); nayereh.soltani@gmail.com (N.S.)

**Keywords:** colloidal Cu@CuAlO_2_-Al_2_O_3_ nanoparticles, polyvinyl pyrrolidone (PVP), gamma radiolytic method, core-shell structure

## Abstract

Colloidal Cu@CuAlO_2_-Al_2_O_3_ bimetallic nanoparticles were prepared by a gamma irradiation method in an aqueous system in the presence of polyvinyl pyrrolidone (PVP) and isopropanol respectively as a colloidal stabilizer and scavenger of hydrogen and hydroxyl radicals. The gamma irradiation was carried out in a ^60^Co gamma source chamber with different doses up to 120 kGy. The formation of Cu@CuAlO_2_-Al_2_O_3_ nanoparticles was observed initially by the change in color of the colloidal samples from colorless to brown. Fourier transform infrared spectroscopy (FTIR) confirmed the presence of bonds between polymer chains and the metal surface at all radiation doses. Results of transmission electron microscopy (TEM), energy dispersive X-ray spectrometry (EDX), and X-ray diffraction (XRD) showed that Cu@CuAlO_2_-Al_2_O_3_ nanoparticles are in a core-shell structure. By controlling the absorbed dose and precursor concentration, nanoclusters with different particle sizes were obtained. The average particle diameter increased with increased precursor concentration and decreased with increased dose. This is due to the competition between nucleation, growth, and aggregation processes in the formation of nanoclusters during irradiation.

## 1. Introduction

Investigation on bimetallic nanoparticles in colloidal solution is of great interest due to their applications in catalysis [1–4], electronics [5], optics [5,6], and the change in the surface plasmon band energy [7,8] relative to the component monometallic particles [9]. Two different types of bimetallic colloids exist: alloys, and those with a core-shell structure [10,11]. In particular, Al-Cu bimetallic nanoparticles have been more recently studied for improving the materials properties of aluminum in terms of increasing hardness or strength associated with decreasing particle size [12]. There are several routes for producing Al-Cu bimetallic nanoparticles, such as laser ablation [13], spark plasma sintering [14], electrochemical deposition [15], self-propagating high-temperature synthesis [16], and radiolytic reduction [12]. Among these, the gamma irradiation induced technique has proven to be an appropriate method for fabrication of monometallic or bimetallic nanoparticles because it produces fully reduced and highly pure metallic nanoparticles, free from by-products and reducing agents [9,17,18]. Large numbers of free electrons and solvated electrons produced during gamma-ray irradiation in colloidal solutions can reduce the metal ions into zero-valent metal atoms without using reducing agents or catalysts and their consequent side reactions [19,20]. In addition, hydroxyl and hydrogen radicals (OH^•^ and H^•^), induced in radiolysis of water are also strong reducing agents in aqueous colloidal solution. To prevent this, the OH^•^ and H^•^ radicals scavenger, isopropanol, was commonly added into the precursor solutions. Isopropanol scavenged OH^•^ and H^•^ radicals and at the same time isopropanol changed into isopropanol radicals, which eventually reduced Al^3+^ and Cu^2+^ into zero-valent atoms of Al^0^ and Cu^0^ as shown in the following reactions [3]:

(1) $${\text{OH}}^{•}+{\text{CH}}_{3}\text{CH}(\text{OH}){\text{CH}}_{3}\to {\text{H}}_{2}\text{O}+{\text{H}}_{3}{\text{CC}}^{•}(\text{OH}){\text{CH}}_{3}$$

(2) $${\text{H}}^{•}+{\text{CH}}_{3}\text{CH}(\text{OH}){\text{CH}}_{3}\to {\text{H}}_{2}+{\text{H}}_{3}{\text{CC}}^{•}(\text{OH}){\text{CH}}_{3}$$

(3) $${\text{Cu}}^{2+}+2({\text{H}}_{3}-{\text{CC}}^{•}(\text{OH}){\text{CH}}_{3})\to 2({\text{CH}}_{3}-\text{CO}-{\text{CH}}_{3})+{\text{Cu}}^{0}+2{\text{H}}^{+}$$

(4) $${\text{Al}}^{3+}+3({\text{H}}_{3}-{\text{CC}}^{•}(\text{OH}){\text{CH}}_{3})\to 3({\text{CH}}_{3}-\text{CO}-{\text{CH}}_{3})+{\text{Al}}^{0}+3{\text{H}}^{+}$$

To prevent increasing particle size, a polymer is often used, either natural or synthetic, with some affinity for metals. The polymer is adsorbed on the cluster in aqueous solution and reduced surface tension [21]. These substances also control both the reduction rate of metal ions and the aggregation process of metal atoms [22]. It was reported that polyvinyl pyrrolidone (PVP) could stabilize colloidal particles in water and many non-aqueous solvents by adsorbing onto a broad range of materials, such as metals (e.g., gold, silver, and iron), and metal oxides (kaolinite, TiO_2_, iron oxide, and alumina) [23,24]. Fourier transform infrared spectroscopy (FTIR) could be used to confirm the presence of bonds between polymer chains and the metal surface.

The purpose of the present work is to report the synthesis of the PVP-capped colloidal Al-Cu bimetallic nanoparticles under the gamma-irradiation method. The textural and morphological characteristics of the prepared Al-Cu bimetallic nanoparticles were studied with various techniques to verify the influence of dose and ion concentrations on morphology and particle size distribution of the nanoparticles, as well as to explore other parameters of interest.

## 2. Results and Discussion

### 2.1. Formation of Al-Cu Clusters in the Presence of PVP

**Figure 1:**
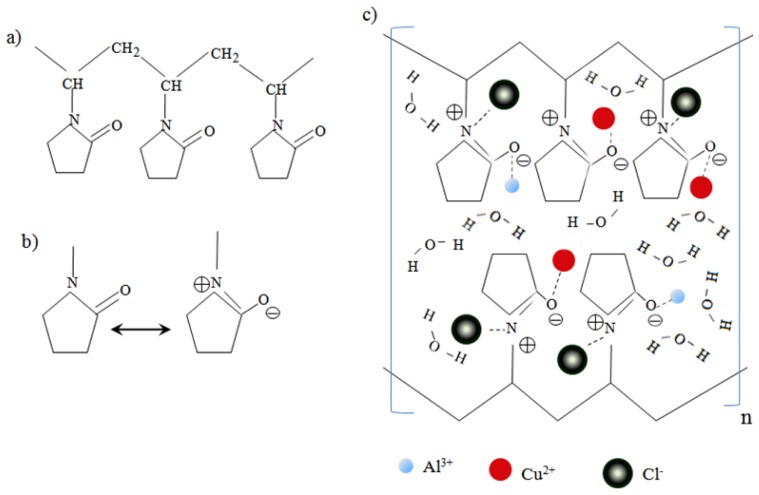
(**a**) A description of one short chain of PVP polymer; (**b**) Resonance structures of a pyrene ring in PVP molecule [23]; (**c**) A proposed oversimplified mechanism of interactions between PVP and metal ions before irradiation.

An oversimplified scheme of the interactions between the PVP capping agent and metal ions before irradiation is shown in Figure 1, which shows that the Al (III) and Cu (II) ions are bound by the ionic bonds between the metallic ions and the amide group in a polymeric chain. PVP acts as a stabilizer for dissolved metallic salts through steric and electrostatic stabilization of the amide groups of the pyrrolidone rings [25]. However, the real mechanism is more complex, due to the presence of hydrogen bonds between water molecules themselves and between the water molecules and the carbonyl polarized groups or positivity nitrogen from the pyrrolidone rings. Moreover, each ion is surrounded with a shell of water molecules [26,27].

The reduction process is controlled by the respective redox potentials [28]. Copper ions (Cu^2+^) are more easily reduced (higher redox potential, E^0^(V) = +0.34) than aluminum ions (Al^3+^, E^0^(V) = −1.66) and for this reason the rate of reaction of solvated electrons in the solution with Cu ions are higher than with Al ions. Thus, when bivalent Cu ions are irradiated, the reduction occurs from the reaction of solvated electrons and possibly H^•^ atoms, in so far as H^•^ atoms are not scavenged by isopropanol, and Cu zero-valent content increases (reaction 5). Then in a further step, when Cu^2+^ ions are depleted, the reduction of Al^3+^ increased, which occurs exclusively at the surface of the Cu particles (reactions 6 and 7) [12]. Accordingly, the resulting particles are bilayered with a core of the more easily reduced metal and a shell made of the other metal.

(5) $${\text{Cu}}^{2+}+2{\text{e}}_{\text{aq}}^{-}\to {\text{Cu}}^{0}\left(\text{or }{\text{Cu}}^{2+}+2{\text{H}}^{•}\to {\text{Cu}}^{0}+2{\text{H}}^{+}\right)\;\;\;,{\text{Cu}}^{0}+{\text{Cu}}^{0}\to {\text{Cu}}_{2}^{0}$$

(6) $${\text{Cu}}_{\text{n}}^{0}+{\text{Al}}^{3+}\to {\left({\text{Cu}}_{\text{n}}\text{Al}\right)}^{3+}+3{\text{e}}_{\text{aq}}^{-}\to {\left({\text{Cu}}_{\text{n}}\text{Al}\right)}^{0}$$

(7) $$\begin{array}{l} {\left({\text{Cu}}_{\text{n}}{\text{Al}}_{\text{m}}\right)}^{\text{x}+}+{\text{Al}}^{3+}\to {\left({\text{Cu}}_{\text{n}}{\text{Al}}_{\text{m}+1}\right)}^{(\text{x}+3)+}+{\text{e}}_{\text{aq}}^{-}\to \\ {\left({\text{Cu}}_{\text{n}}{\text{Al}}_{\text{m}+1}\right)}^{(\text{x}+2)+}\cdots \to {\left({\text{Cu}}_{\text{n}}{\text{Al}}_{\text{m}+1}\right)}^{0}\to \text{Al}(\text{shell})-\text{Cu}(\text{core}) \end{array}$$

**Figure 2:**
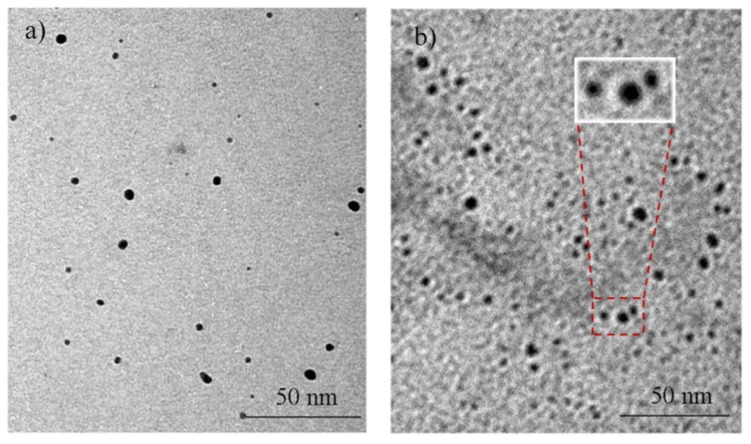
TEM images of (**a**) Cu nanoparticles and (**b**) Cu@CuAlO_2_-Al_2_O_3_ nanoparticles.

It is a well known fact that to prepare pure Cu, Al, or Al-Cu bimetallic nanoparticles by radiation synthesis in air without the influence of oxygen is not easy. The intended Al-Cu bimetallic nanoparticles are contaminated with oxygen. Therefore, it is safe to note that the final product of this synthesis would be Cu@CuAlO_2_-Al_2_O_3_ nanoparticles as we can see later from the EDX and XRD analyses. Figure 2 shows the comparison between TEM images of Cu nanoparticles and the core-shell structure of Cu@CuAlO_2_-Al_2_O_3_ nanoparticles. In a TEM image (Figure 2b), the shells can be partially distinguished from the cores, because the lower atomic number of Al provides less contrast in a TEM image than Cu. The boundary between the core and shell is not sharp, however, which suggests that the shells might not be pure Al, but CuAlO_2_ and Al_2_O_3_ instead. The same interpretation reported for other elements by M. S. Shore [29].

**Figure 3:**
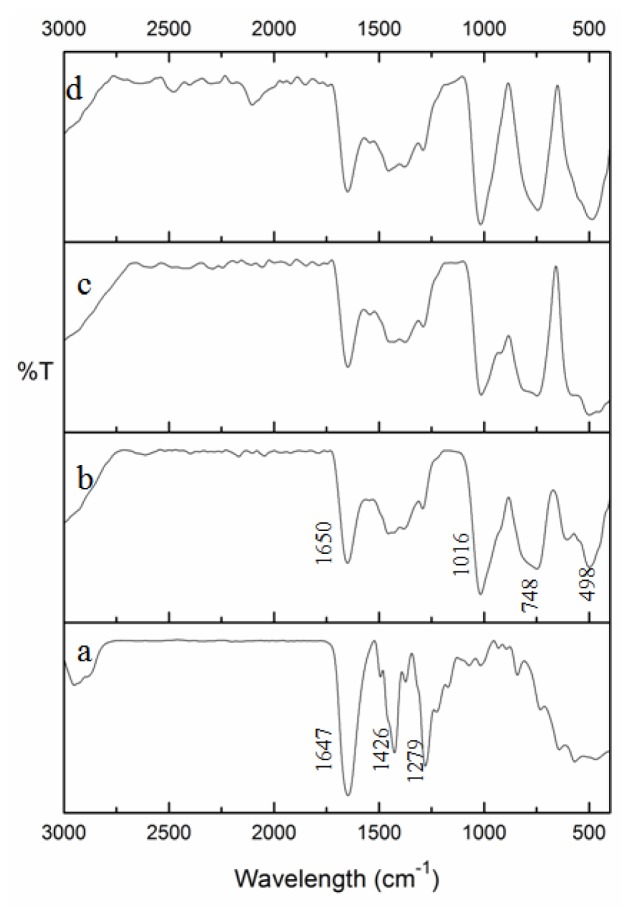
FT-IR spectra of (**a**) pure PVP, and PVP-capped Cu@CuAlO_2_-Al_2_O_3_ nanoparticles at: (**b**) 80; (**c**) 100; and (**d**) 120 kGy radiation dose.

Figure 3 presents a comparison of FT-IR spectra of PVP alone and PVP-capped Cu@CuAlO_2_-Al_2_O_3_ nanoparticles at doses of 80, 100, and 120 kGy. The spectra are given in the range 3000–400 cm^−1^, because the main chemical changes occurred in this range. In the spectrum of pure-PVP (Figure 3a), the peaks located at 1647, 1426, and 1279 cm^−1^, are assigned to the C=O stretching vibration, CH_2_ bending vibration, and C-N stretching vibration band, respectively [30–33]. The PVP molecules in aqueous solution may take resonance structures as shown in Figure 1b [24]. In the FT-IR spectrum of PVP-capped Cu@CuAlO_2_-Al_2_O_3_ nanoparticles (Figure 3b), compared to pure PVP (Figure 3a), the intensity of the C=O stretching band decreased, indicating on the formation of intermolecular bonds between PVP and shell. As shown in Figure 3b, the adsorption band appeared at 1016 cm^−1^ generally assigned to C-N, indicating the coordination between N and Cu@CuAlO_2_-Al_2_O_3_ nanoparticles. Compared to pure PVP, this peak was reduced due to the strengthened C–N bonds of the pyridine when the metal was incorporated. The change of the peak shape below 900 cm^−1^ is associated with the Al-O bond vibrations [34,35]. These peaks slightly increased with increasing dose, indicating the amount of that the oxide form of Al on the surface of Cu nanoparticles increased (Figure 3b–d).

### 2.2. Effect of Dose

**Figure 4:**
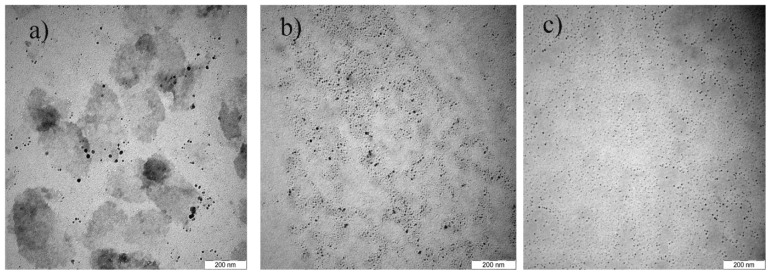
TEM image of colloidal Cu@CuAlO_2_-Al_2_O_3_ nanoparticles at: (**a**) 80 kGy with average size 12 nm; (**b**) 100 kGy with average size 6 nm; and (**c**) 120 kGy with average size 4.5 nm.

The morphology of the Cu@CuAlO_2_-Al_2_O_3_ colloidal nanoparticles was observed by employing the TEM technique. The shape of the particles in all colloids was quasi-spherical. Figure 4 shows a representative TEM image of the PVP-capped Cu@CuAlO_2_-Al_2_O_3_ nanoparticles at various radiation doses. It appears that the Cu@CuAlO_2_-Al_2_O_3_ particles are well separated with no agglomeration tendency, which is roughly parallel to the stability of the colloids.

The average size of Cu@CuAlO_2_-Al_2_O_3_ nanoparticles decreases from 12 nm at 80 kGy to 4.5 nm at 120 kGy radiation dose. Variation in the particle size could refer to the difference in the nucleation and growth processes. At low doses, the reduction rate was slow and only small numbers of nuclei were formed. So the number of nuclei remained constant or increased slower than the amount of unreduced ions. The unreduced ions collided with the nuclei already formed that led to the formation of larger ions and subsequently after interacting with solvated electrons produced larger particles. At higher doses, the enhanced reduction rate favored the generation of many more nuclei. When the number of nuclei increased faster than that of unreduced ions, little ion association can take place and smaller particles would be obtained [36].

**Figure 5:**
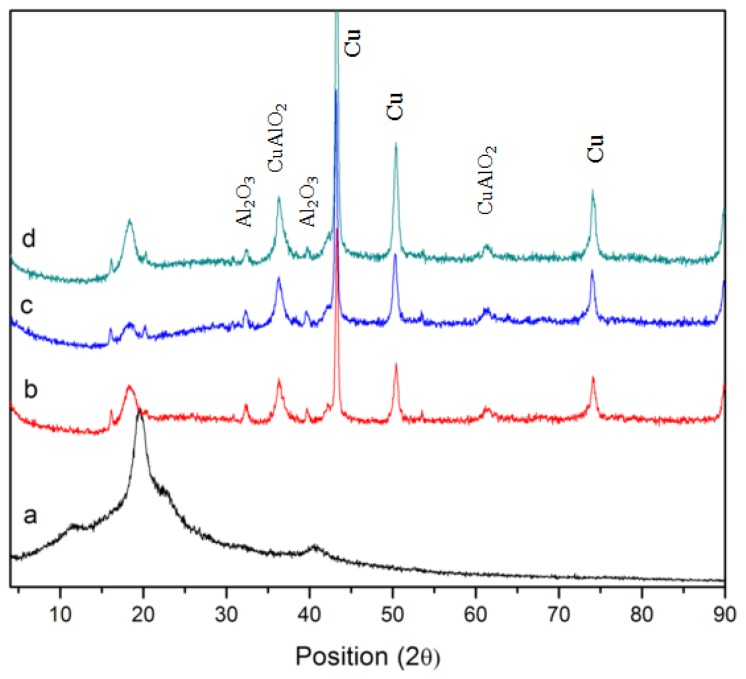
X-ray diffraction patterns of: (**a**) pure PVP, PVP-capped Cu@CuAlO_2_-Al_2_O_3_ nanoparticles at: (**b**) 80; (**c**) 100; and (**d**) 120 kGy radiation dose.

XRD patterns of pure PVP and PVP-capped Cu@CuAlO_2_-Al_2_O_3_ nanoparticles at various radiation doses are presented in Figure 5. In Figure 5b–d, diagrams exhibit reflections that could clearly be assigned to the typical face-centered cubic (fcc) Cu pattern, that increased by increasing dose. In addition, Al_2_O_3_ and CuAlO_2_ reflections were found in the samples. Low intensities of CuAlO_2_ and Al_2_O_3_ diffraction peaks are assumed to arise from the fact that Al clusters are thinly coated on the Cu nanoparticles. The oxidation form of Al may result in a slight inevitable surface oxidation during the process of PVP-capping, washing, or drying. This result is in good agreement with the EDX results of samples.

**Figure 6:**
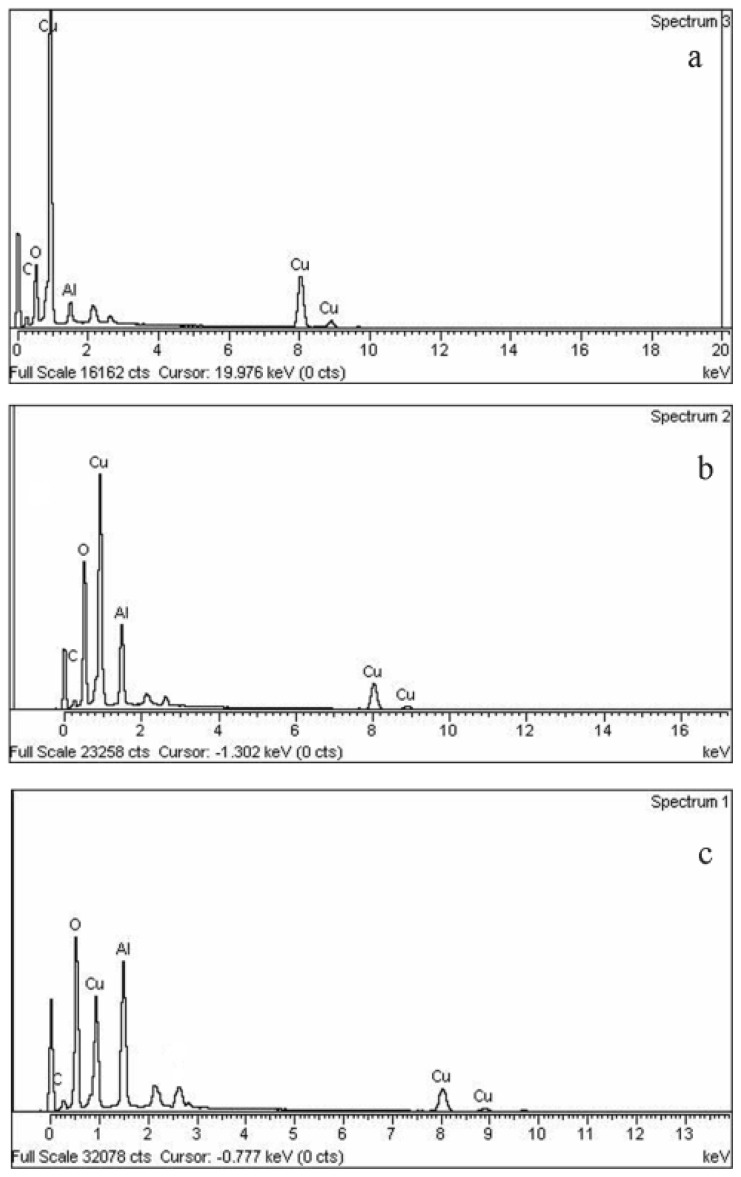
EDX spectrum of PVP-capped Cu@CuAlO_2_-Al_2_O_3_ nanoparticles at: (**a**) 80; (**b**) 100; and (**c**) 120 kGy radiation dose.

The quantitative determination of the Al and Cu contents, on the surface of the samples, was made by EDX analysis (Figure 6). At low dose, the reduction starts by reducing copper ions to zerovalent atoms as far as Cu^0^ atoms were more concentrated than Cu^2+^ ions. In further steps by increasing dose, the reduction of Al^3+^ increased, which was eventually assaulted by oxygen at the surface of the Cu particles. The existence of a carbon peak confirms the presence of PVP stabilizer in the prepared EDX samples.

### 2.3. Effect of Initial Ion Concentration

**Figure 7:**
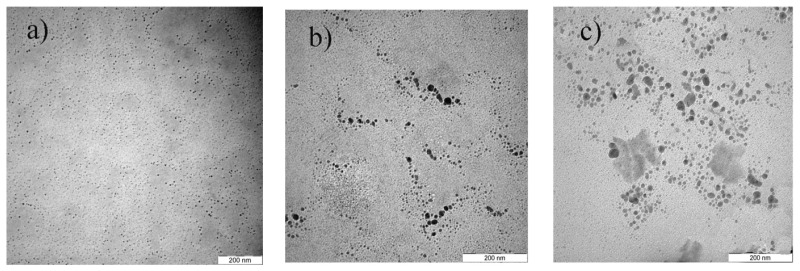
TEM images of Cu@CuAlO_2_-Al_2_O_3_ nanoparticles with total ion concentration of: (**a**) 5.0 × 10^−5^ mol/mL and average size of 4.5 nm; (**b**) 5.7 × 10^−5^ mol/mL and average size of 10 nm; and (**c**) 6.4 × 10^−5^ mol/mL and average size of 13 nm at 120 kGy.

The effect of precursor concentration on the formation of Cu@CuAlO_2_-Al_2_O_3_ nanoparticles was studied with varying concentrations of total ions: 5.0 × 10^−5^, 5.4 × 10^−5^, 5.7 × 10^−5^, 6.0 × 10^−5^, and 6.4 × 10^−5^ mol/mL at a constant concentration of PVP and at the fixed Al/Cu mole ratio of 70/30. In this mole ratio, the most probable case is the existence of a uniform core-shell structure of Cu@CuAlO_2_-Al_2_O_3_. Figure 7 shows the TEM results of the Cu@CuAlO_2_-Al_2_O_3_ nanoparticles obtained at various AlCl_3_ and CuCl_2_ concentrations. Only the TEM images for ion concentrations of 5.0 × 10^−5^, 5.7 × 10^−5^, and 6.4 × 10^−5^ mol/mL at 120 kGy are shown here. TEM results show the average size of Cu@CuAlO_2_-Al_2_O_3_ nanoparticles increases from 4.5 nm at the lowest ion concentration to 13 nm at the highest.

**Figure 8:**
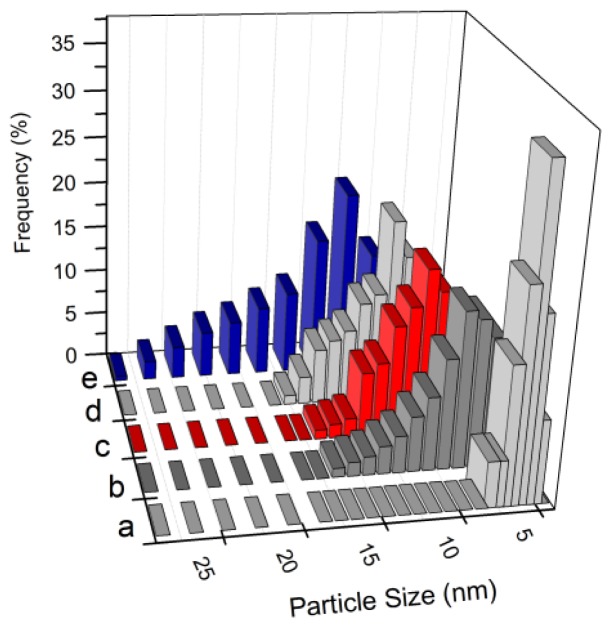
Particle size distribution of Cu@CuAlO_2_-Al_2_O_3_ nanoparticles at 120 kGy and for various concentrations: (**a**) 5.0 × 10^−5^; (**b**) 5.4 × 10^−5^; (**c**) 5.7 × 10^−5^; (**d**) 6.0 × 10^−5^; and (**e**) 6.4 × 10^−5^ mol/mL.

Increasing ion concentration causes the bimetallic nanoparticles to become larger in size, which can be seen by shifting the center of size distribution in Figure 8 towards a larger size. Three main reasons describe these behaviors of nanoparticles. Firstly, by increasing concentration of metal ions in the solution, the rate of ion association under irradiation to form larger particles increases. Secondly, the small particles make wavy movements and collide with each other in solution, leading to particle aggregation. On the other hand, the viscosity of the aqueous solution and therefore speed of particle movement can be changed by the ratio of polymer/ions. Increasing the ion concentration (decreasing ratio of polymer/ions) can increase the collision probability. Finally, the adsorption of PVP on the surface of nanoparticles can reduce the surface energy and further agglomeration of nanoparticles [37,38]. Thus, increasing ion concentration reduces the tendency of polymer capping on the surface of nanoparticles, leading to larger particles.

Composition analysis for each ion concentration of Cu@CuAlO_2_-Al_2_O_3_ by EDX is summarized in Table 1. Results of EDX show that, under the same radiation dose by increasing ion concentration, concentration of reduced Al atoms increases. The atomic percentages of Al nanoparticles are always higher than that of Cu nanoparticles, indicating that the Cu core was mostly covered by an Al layer.

**Figure 9:**
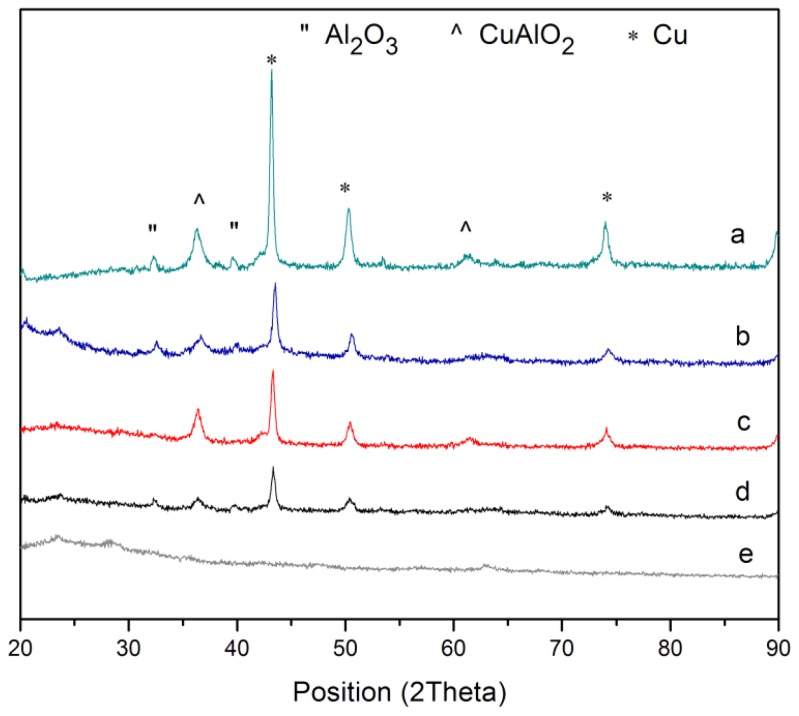
XRD patterns of Cu@CuAlO_2_-Al_2_O_3_ nanoparticles at 120 kGy for various ion concentrations: (**a**) 5.0 × 10^−5^; (**b**) 5.4 × 10^−5^; (**c**) 5.7 × 10^−5^; (**d**) 6 × 10^−5^; and (**e**) 6.4 × 10^−5^ mol/mL.

X-ray powder diffraction diagrams of Cu@CuAlO_2_-Al_2_O_3_ colloids, presented in Figure 9. As can be observed, at lower precursor concentration of zero-valent state copper and alumina reflections was found. The alumina layer mostly covered the Cu core, thus protecting it from corrosion. The oxidation of Al may results in a slight inevitable surface oxidation during the process of washing and drying. By increasing ion concentration, the thickness of the alumina layer that coated the Cu core increased, which can affect the crystallinity of Cu. Therefore, the count of reflection peaks of Cu decreases with increasing ion concentration. The XRD diagram for sample with the highest ion concentration clearly shows that the sample is entirely amorphous (Figure 9e).

## 3. Experimental Section

### 3.1. Materials

Copper chloride dihydrate (CuCl2·2H2O) and aluminum chloride hexahydrate (AlCl3·6H2O), which are used as precursors, were purchased from Systerm. PVP (MW = 29,000, Sigma Aldrich, St Louis, MO, USA) and isopropanol were used as a capping agent to reduce the agglomeration and radical scavenger of hydroxyl radicals, respectively. The precursors, capping agent, and radical scavenger were used “as received”, without further purification.

### 3.2. Preparation of PVP-Capped Cu@CuAlO_2_-Al2O_3_ Nanoparticles

In order to make PVP-capped Cu@CuAlO_2_-Al_2_O_3_ nanoparticles, we used a stock solution of 3% PVP by dissolving PVP powder in distilled water, then AlCl3 and CuCl2 were added into the PVP solution at constant Al/Cu mole ratio (Al/Cu = 70/30) in the presence of isopropanol (1.3 mol/L). The solution was magnetically stirred, bubbled with nitrogen for 1 h at 70 °C, and the solution divided into several parts in the glass tubes. The mixture solution was irradiated by Co-60 γ-ray source chamber with a dose rate of 2.9 kGyh^−1^ and each sample received different doses of 80, 100, and 120 kGy. In this method, γ-rays interact with matter in the solution, mainly by photoelectric absorption and Compton scattering to reduce secondary electrons which induced reactive species, such as solvated electrons. These electrons reduce the metal ions to metal atoms, which then aggregated to form metallic nanoparticles.

### 3.3. Characterization

FT-IR spectra were recorded using a PerkinElmer FTIR model 1650 spectrometer. The particle size and particle distribution were determined from transmission electron microscopy (TEM) micrographs (HITACHI H-7100 TEM). Samples for TEM studies were prepared by placing a drop of the irradiated solutions on a TEM copper grid. The sample on the grids was allowed to dry naturally for several hours. The TEM characterization was carried out at 100 keV. The microstructure of the Al-Cu nanoparticles was characterized by the XRD technique using a Shimadzu diffractometer (Model XRD 6000) using Cu Kα (0.154 nm) X-rays to generate diffraction patterns. Energy dispersive X-ray analysis (EDX-JSM-6400 JEOL) was used to determine the composition of the bimetallic nanoparticles and their element weight percentage. For the preparation of EDX, XRD and FTIR samples, colloidal samples were washed several times with water and ethanol by centrifugation and finally dried at 50 °C at atmospheric pressure to make a fine powder.

## 4. Conclusions

We have succeeded in synthesizing Cu@CuAlO_2_-Al_2_O_3_ nanoparticles in core-shell structure from a CuCl2 and AlCl3 solution stabilized in PVP by a gamma radiolytic technique. The FT-IR analysis of colloidal nanoparticles shows that PVP was absorbed onto the Al shell during particle growth. The particle size was determined by using transmission electron microscopy. At high doses and low ion concentration, where the nucleation event is very fast, the radiation synthesis produced smaller sizes of nanoparticles. At lower doses and higher ion concentration, where the nucleation event is less than the unreduced ions, the radiation synthesis produced larger sizes of nanoparticles following aggregation. Consequently, at a fixed PVP, capping the Cu@CuAlO_2_-Al_2_O_3_ nanoparticle growth process is a dose and precursor concentration dependent process.

## Figures and Tables

**Table 1:** Results of EDX for Cu@CuAlO_2_-Al_2_O_3_ nanoparticles in various ion concentrations at 120 kGy and fixed precursor mole ratio of Al/Cu = 70/30.

Precursor ion concentration (mol/mL)	Al content after irradiation (atomic %)	Cu content after irradiation (atomic %)
5.0 × 10^−5^	64	36
5.4 × 10^−5^	83.19	16.81
5.7 × 10^−5^	86.8	13.2
6.0 × 10^−5^	86.9	13.1
6.4 × 10^−5^	87.48	12.52
